# A PZT-Based Electromechanical Impedance Method for Monitoring the Soil Freeze–Thaw Process

**DOI:** 10.3390/s19051107

**Published:** 2019-03-05

**Authors:** Jicheng Zhang, Chuan Zhang, Jiahao Xiao, Jinwei Jiang

**Affiliations:** 1 School of Urban Construction, Yangtze University, Jingzhou 434023, China; zhangjicheng@yangtzeu.edu.cn (J.Z.); 201772373@yangtzeu.edu.cn (J.X.); 2 Key Laboratory of Transportation Tunnel Engineering, Ministry of Education, Southwest Jiaotong University, Chengdu 610031, China; 3 Smart Material and Structure Laboratory, Department of Mechanical Engineering, University of Houston, Houston, TX 77204, USA

**Keywords:** Lead Zirconate Titanate (PZT), smart aggregates, soil freeze–thaw process, electro-mechanical impedance (EMI) method

## Abstract

It is important to conduct research on the soil freeze–thaw process because concurrent adverse effects always occur during this process and can cause serious damage to engineering structures. In this paper, the variation of the impedance signature and the stress wave signal at different temperatures was monitored by using Lead Zirconate Titanate (PZT) transducers through the electromechanical impedance (EMI) method and the active sensing method. Three piezoceramic-based smart aggregates were used in this research. Among them, two smart aggregates were used for the active sensing method, through which one works as an actuator to emit the stress wave signal and the other one works as a sensor to receive the signal. In addition, another smart aggregate was employed for the EMI testing, in which it serves as both an actuator and a receiver to monitor the impedance signature. The trend of the impedance signature with variation of the temperature during the soil freeze–thaw process was obtained. Moreover, the relationship between the energy index of the stress wave signal and the soil temperature was established based on wavelet packet energy analysis. The results demonstrate that the piezoceramic-based electromechanical impedance method is reliable for monitoring the soil freezing and thawing process.

## 1. Introduction

As one of the most important construction materials for engineering structures, soil is irreplaceable in civil engineering. In addition to earthquakes and soil erosion, soil expansion and contraction induced by the periodic freeze–thaw process is also a cause of fatal damage to engineering structures such as roads, bridges, and buildings.

However, the soil freezing and thawing process can not only cause soil expansion and contraction but can also change the soil’s mechanical properties. In recent decades, numerous studies about the influences of the freeze–thaw process on soil mechanical properties have been reported. Aldaood et al. [1] demonstrated that the effect of freezing–thawing cycles on the durability of gypsum-containing soil is severe by comparing the uncompressed compressive strengths of gypsum soils with different gypsum contents under freezing–thawing cycles. Qi et al. [2] analyzed the changes in the density, strength, and resilient modulus of Lanzhou loess soil under different freezing conditions. Salour et al. [3] investigated pavement structural behaviors during the spring thaw by using a falling weight deflectometer. Yang et al. [4] studied the seasonal frost effects on the dynamic behavior of a twenty-story office building. Simonsen et al. [5] analyzed the effects of freezing–thawing soil on pavement by using the Finite Element Modeling (FEM) method. Simonsen et al. [6] further studied the influences of the soil type, permeability, drainage conditions, and thawing rate on the thaw weakening of pavement structures in cold regions. Guymon et al. [7] developed a mathematical model that can be used to estimate the frost heave and thawing subsidence in various situations. Shoop et al. [8] proposed a material model for thawing soil behavior through the FEM method and validated the numerical model by conducting direct shear tests on thawed soil samples. Graham et al. [9] studied the effect of the freeze–thaw cycle on the stress state of clay soil.

Generally, evaluation of the soil freeze–thaw process is mainly performed through on-site investigation. This method is costly and requires a great deal of manpower and material resources. Nowadays, emerging microwave detection methods have already been successfully utilized as a promising approach to monitor the soil freezing–thawing status. Mcdonald et al. [10] outlined the application and principles of microwave remote sensing technology in the estimation of surface freeze–thaw states. Chai et al. [11] proposed a new method to determine the freezing and thawing degree based on the use of passive microwave remote sensing technology. Zwieback et al. [12] presented a fusion algorithm to determine freeze–thaw status by combination of Ku-band and C-band scatterometer data. Zhang et al. [13,14,15] researched the freezing–thawing status, duration, and area of near-surface soils in the United States by using passive microwave remote sensing technology. Han et al. [16] detected the springtime thawing of near-surface soil in northern China using passive and active microwave remote sensing technology. Wu et al. [17] monitored the freezing–thawing process of soil by utilizing a Global Position System (GPS) interference reflectometer. Jadoon et al. [18] utilized Ground-Penetrating Radar (GPR) to monitor soil freezing–thawing cycles. Judge et al. [19] performed the freeze–thaw classification of prairie soils by using the Special Sensor Microwave/Imager (SSM/I) radiobrightnesses. Zhao et al. [20] presented a newly developed algorithm for distinguishing the freeze–thaw status of surface soil based on the Advanced Microwave Scanning Radiometer—Enhanced (AMSR-E) which records brightness temperature in the afternoon and after midnight. Also, the NASA scatterometer [21,22,23], CT techniques [24,25,26,27,28,29,30], and nuclear magnetic resonance techniques [31,32,33] have been used to analyze the characteristics of frozen soil.

Lead Zirconate Titanate (PZT), a type of piezoceramic material, has been extensively applied in structure health monitoring (SHM) [34,35,36,37] and damage detection [38,39] in the past few decades. Compared with non-destructive testing (NDT) devices, the piezoceramic-based transducer has the advantages of small size, high sensitivity, fast response, wide bandwidth, and low cost, and piezoceramic transducers can also be used for structural impact monitoring [40,41,42,43,44,45]. Since the PZT material has both direct and reverse piezoelectric effects, it can serve as both an actuator and a sensor. Nowadays, there are mainly two types of methods for SHM using PZT-based transducers, namely, the active sensing method [46,47,48] and the electromechanical impedance method [49,50,51,52]. Based on these two methods, PZT transducers have been employed in numerous studies. Soh et al. [53] demonstrated the feasibility of the PZT-based transducer to monitor a typical reinforced concrete (RC) bridge. Visalakshi et al. [54] compared the performances of embedded and surface-bonded PZT patches in the corrosion detection of RC structures. Feng et al. [55] monitored different types of damage to concrete piles by using PZT-based transducers. PZT transducers have been used to detect damage to an oil pipeline [56,57,58] and a concrete-filled steel tube (CFST) [59,60]. Karayannis et al. [61] conducted a damage evaluation of rebar inside concrete by using PZT transducers. Moreover, PZT transducers have also been researched in other fields such as timber damage and moisture detection [62,63,64,65,66], SHM of aerospace structures [67,68,69] and mechanical components [70,71,72,73], and wind turbine monitoring [74,75,76,77]. Song et al. [78,79,80,81,82] adopted piezoceramic-based smart aggregates, which have been verified to be more stable and reliable, to monitor cement strength development and to test the dynamic behaviors of concrete structures subject to seismic excitation.

In this paper, several PZT-based smart aggregates were employed to monitor the soil freezing–thawing process through the EMI method and the active sensing method. Through experimental research, the changes in the resonance peaks in the EMI spectra over temperature were measured and analyzed; this demonstrated itself to be an effective indicator to characterize the soil freeze–thaw process. Also, the relationship between the soil freezing–thawing status and the EMI signature was established. Additionally, the wavelet packet energy index was used to quantify the soil freezing–thawing status, which verified the effectiveness of the EMI method in monitoring the soil freeze–thaw process.

## 2. Principles

### 2.1. Electromechanical Impedance Method

**Figure 1:**
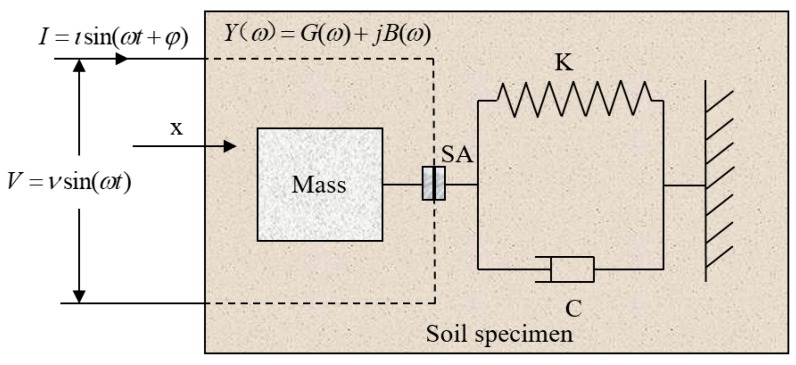
The electromechanical impedance model of a Lead Zirconate Titanate (PZT)-driven structural system.

Due to its direct and reverse piezoelectric effects, the PZT transducer can be used as both an actuator and a receiver. The one-dimensional model that illustrates the coupling between the PZT transducer and the host structure is displayed in Figure 1. In this system, when alternating current is applied to the PZT transducer, it will create harmonic vibrations with high frequencies which will further drive the vibrations of the host structure and induce a structural response in the form of electromechanical impedance. Any changes in the mechanical properties of the host structure will lead to variations in the electromechanical impedance.

Equation (1) is the mathematical expression used to illustrate the relationship between the electric admittance (the reciprocal of impedance) and the frequency based on the piezoelectric wave equation [83]:

(1) $$Y=i\omega a\left[\varepsilon_{33}^{T}\left(1-i\delta \right)-\frac{Z\left(\omega \right)}{Z\left(\omega \right)+Z_{A}\left(\omega \right)}{\left(d_{32}\right)}^{2}Y_{22}^{E}\right]$$

where a is the geometric constant of the PZT driver, i is an imaginary unit, ω is the angular frequency of the applied current, δ is the dielectric loss factor, $\varepsilon_{33}^{T}\left(1-i\delta \right)$ is the complex permittivity without stress, Z is the mechanical impedance of the structure, $Z_{A}$ is the mechanical impedance of the PZT material, $d_{32}$ is the piezoelectric constant, and $Y_{22}^{E}$ is the Young’s modulus of the PZT at zero electric field.

According to IEEE Std. 176-1987 [84,85], $c_{22}^{D}$ and $k_{t}$ can be determined using the following equations:

$$c_{22}^{D}=4\rho L^{2}f_{p}$$

$$k_{t}=\frac{\pi }{2}\frac{f_{s}}{f_{p}}\tan\left(\frac{\pi }{2}\frac{f_{p}-f_{s}}{f_{p}}\right)$$

where $c_{22}^{D}$ and $k_{t}$ represent the elastic stiffness constant at constant electric displacement and the electromechanical coupling constant, respectively; $f_{p}$ and $f_{s}$ are the frequencies at which the real parts of the impedance Z and the admittance Y, respectively, have a maximum. Both $f_{p}$ and $f_{s}$ can be measured by the impedance analyzer; thus, based on the above equations, $c_{22}^{D}$ and $k_{t}$ can be obtained. Furthermore, by substituting the two calculated parameters into the following basic equations [85], $\varepsilon_{33}^{T}$, $d_{32}$, and $Y_{22}^{E}$ can be acquired:

$$k_{t}^{2}=\frac{d_{32}^{2}}{\varepsilon_{33}^{T}c_{22}^{D}}$$

$$c_{22}^{D}=c_{22}^{E}/\left(1-k_{t}^{2}\right),\;Y_{22}^{E}=1/c_{22}^{E}$$

$$\varepsilon_{33}^{T}=C\frac{l}{A}$$

where l and A denote the thickness and area of the PZT patch, respectively. C represents the capacitance, which can be measured by a capacitor.

### 2.2. Active Sensing Method

The active sensing method is adopted to monitor the soil freezing–thawing process as well. In this method, two PZT-based smart aggregates are embedded at pre-determined locations inside the soil specimen. Between them, one serves as an actuator to emit a stress wave signal, and the other one is used as a receiver to capture the signal. Since the soil specimen acts as a medium for the transmission of the signal, the received signal directly depends on the soil’s physical properties. As the soil sample experiences the freeze–thaw process, its properties will change. This will further influence the propagation of the signature through the soil specimen. Hence, the soil freeze–thaw status can be evaluated by measuring the signal variations with temperature.

### 2.3. Wavelet Packet Analysis

Wavelet packet analysis is based on wavelet analysis, but it maintains a higher time–frequency resolution than wavelet analysis [86,87]. In addition, more suitable frequency bands can be chosen to match the spectra of the signal through this method. Previously, wavelet packet analysis has been used to analyze the active sensing data for SHM and damage detection [88,89]. Based on the active sensing method, wavelet packet analysis has also been performed to quantitatively describe the soil status during the freeze–thaw process [90,91,92].

The signal *S* collected by the piezoceramic-based transducer can be decomposed by an *n*-layer wavelet packet, and its mathematical expression can be written as

(2) $$S=s_{1}+s_{2}+\ldots +s_{i}+\ldots +s_{2^{n-1}}+s_{2^{n}}$$

where S is the original signal and $s_{i}$ is the corresponding decomposed subsignal to each frequency band.

Representing $S_{i}$ in the form of a vector gives

(3) $$S_{i}=\left[s_{i,1},s_{i,2},s_{i,3},\ldots ,s_{i,m-1},s_{i,m}\;\right]$$

where i stands for the frequency band and m is the total number of collected data samples.

The sub-signal energy vector in each frequency band of the *n*-layer signal can be defined as

(4) $$\bar{E}=\left[e_{1},e_{2},e_{3},\ldots ,e_{i},\ldots ,e_{2^{n-1}},e_{2^{n}}\right]$$

where e is the sub-signal energy in each band of the *n*-layer signal. $e_{i}$ can be expressed as

(5) $$e_{i}=\overset{n}{\underset{k=1}{\sum }}{\left|x_{i,k}\right|}^{2}$$

where $x_{i,k}$ represents the signal data at the frequency band *i*.

Therefore, the energy of the signal decomposed by the *n*-layer wavelet packet can be represented by the sum of the signal energy vectors of each frequency band, namely,

(6) $$E=\overset{2n}{\underset{k=1}{\sum }}e_{i}$$

Based on the above wavelet packet analysis, the soil freezing–thawing status can be assessed by analyzing the energy of the stress wave signal received by the PZT transducer. The value of the energy increases as the temperature drops in the freezing process. Conversely, the energy value decreases as the temperature goes up in the defrosting process.

## 3. Experimental Investigation

### 3.1. Experiment Materials

**Figure 2:**
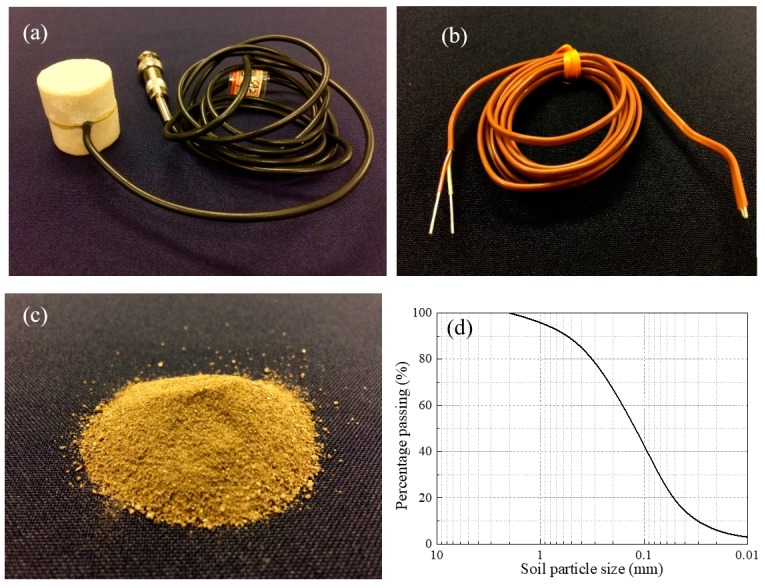
Experimental materials: (**a**) PZT-based smart aggregate; (**b**) K-type thermal transducer; (**c**) Soil sample; (**d**) Soil particle size distribution.

The involved experimental materials are exhibited in Figure 2. In this experiment, smart aggregate with a PZT patch sandwiched between two marble cylinders was adopted, as shown in Figure 2a. A K-type thermocouple sensor was used to monitor the temperature change in the freeze–thaw process, as exhibited in Figure 2b. The experimental soil sample was regular surface clay soil collected from a construction site in Houston, USA. The soil was fully dried in a heated drying oven, as shown in Figure 2c, and its particle size distribution curve is displayed in Figure 2d.

### 3.2. Experimental Setup

**Figure 3:**
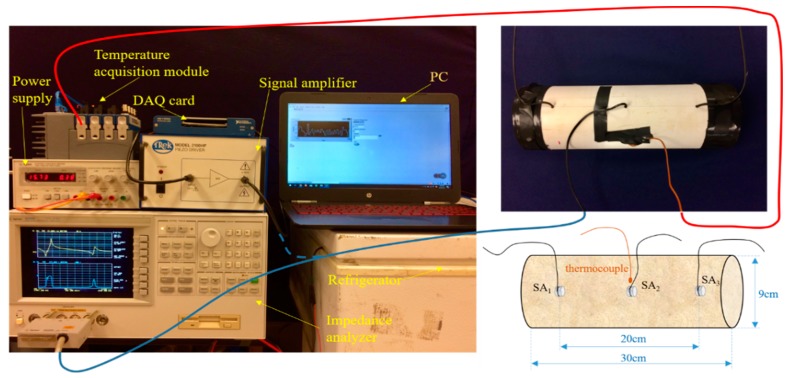
Experimental setup.

The experimental setup is displayed in Figure 3. The soil was encapsulated in a polyvinyl chloride (PVC) tube with a diameter of 9.0 cm and a total length of 30.0 cm. Three smart aggregates (SAs) and one K-type thermocouple sensor were embedded at pre-determined locations in the PVC tube. SA1 and SA3 were embedded 10.0 cm away from opposing ends of the pipe, as shown in Figure 3. SA2 and the thermocouple sensor were placed at the center of the tube. In this research, SA1 was used as an actuator and SA3 was employed as a receiver to monitor the stress wave signal from SA1, the received data was acquired with a DAQ (Data Acquisition) card. In addition, SA2 was used for the EMI testing and served as both a driver and a receiver.

### 3.3. Experimental Procedures

In this experiment, to monitor the soil freeze–thaw process, the soil specimen was placed in a temperature-adjustable refrigerator with a temperature range from room temperature (about 20 °C) to a minimum temperature of −25 ℃. Once the temperature reached −20 °C, the freezer was immediately turned off. The experiment then shifted to the thawing process, during which the temperature gradually rose back to room temperature (around 20 °C). Two aggregates (SA1 and SA3) were adopted in the active sensing method and one aggregate (SA2) was utilized in the EMI testing, and the temperature of the specimen was monitored by the K-type thermocouple sensor. The temperature was recorded every 5 min. In the active sensing testing, SA1 emitted a signature under the excitation of a sweep sine signal with a sweep period of 1.0 s, and the frequency of the sweep sine wave increased linearly from 0.1 to 30 kHz. SA3 served as a receiver to capture the signal. In the EMI testing, SA2 acted as both an actuator and a receiver to acquire the impedance signal, which was obtained by scanning the transducer over a frequency range from 50 kHz to 450 kHz.

What calls for special attention is the fact that temperature changes may have some influence on the captured impedance signals during the experiment due to the long-term depolarization effect of the piezoelectric material. However, for the situation in this study, the temperature is not low enough to cause significant depolarization. This is supported by some studies [93,94] which have shown that the real part of the signal of PZT patches is negligibly affected by the temperature while the imaginary part of the signal may be affected depending on how significantly the temperature changes. This was also verified by a pre-test on a free PZT patch under the temperatures of 20 °C and −20 °C; the experimental results did not show clear changes in the impedance signals. Therefore, in this study, the effect of the temperature change range on the results was limited and can be ignored.

## 4. Results and Analysis

### 4.1. Temperature Measurement during the Freeze–Thaw Process

**Figure 4:**
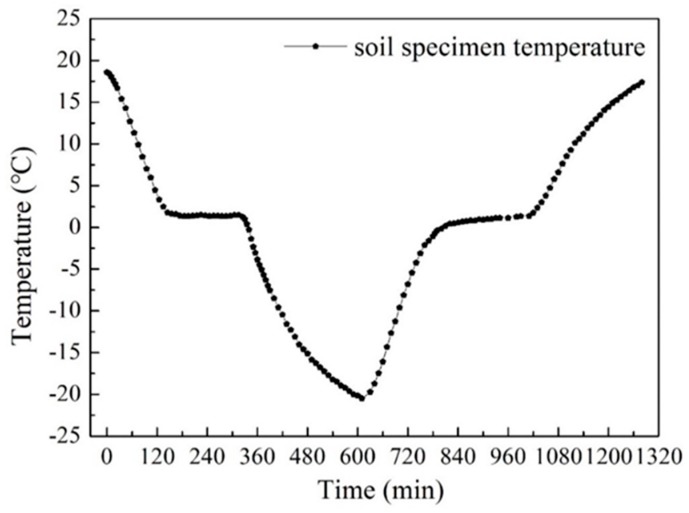
Soil temperature with variation of time.

As shown in Figure 4, the temperature of the specimen was monitored by the thermocouple sensor throughout the freezing–thawing process. The initial temperature of the soil specimen was about 20 °C and the lowest measured value was around −20 °C. Soon after the test started, the temperature of the soil specimen began to decline sharply. It was almost 120 min before the temperature dropped to 0 °C, which was then followed by a freezing process for about 210 min. During this period, the temperature remained nearly unchanged. The reason for this phenomenon is that phase transition occurs during this period, in which the soil moisture gradually turns into ice. This would continuously extract heat, keeping the soil temperature from decreasing. After that, the temperature continued to decrease until it finally reached the pre-set temperature of −20 °C. The entire freezing process lasted about 600 min. During the thawing process, converse results were observed. In addition, a similar phenomenon of the temperature remaining unchanged at temperatures around 0 °C was also observed, and the process lasted approximately 180 min. During this period, the frozen soil gradually defrosted. Upon complete thawing, the duration of the soil thawing process was almost the same as the freezing time. The entire freeze–thaw test lasted about 1320 min.

### 4.2. Impedance Variation during the Freeze–Thaw Process

**Figure 5:**
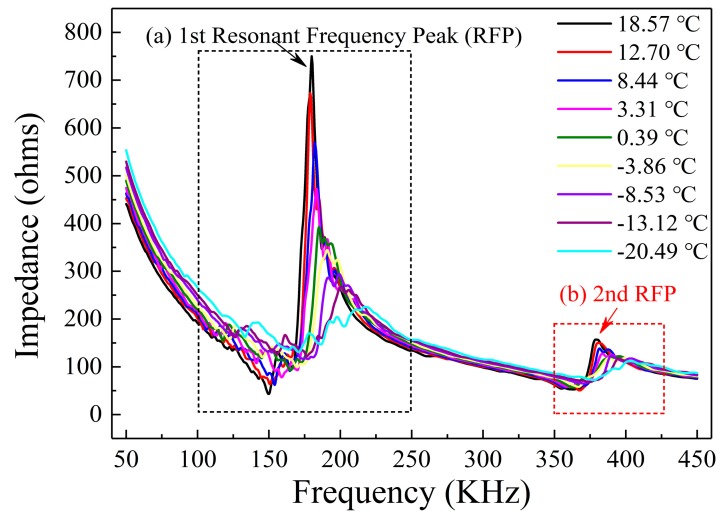
Impedance variation during the soil freezing process: (**a**) the first observed resonant frequency peak (RFP); (**b**) the second observed resonant frequency peak.

**Figure 6:**
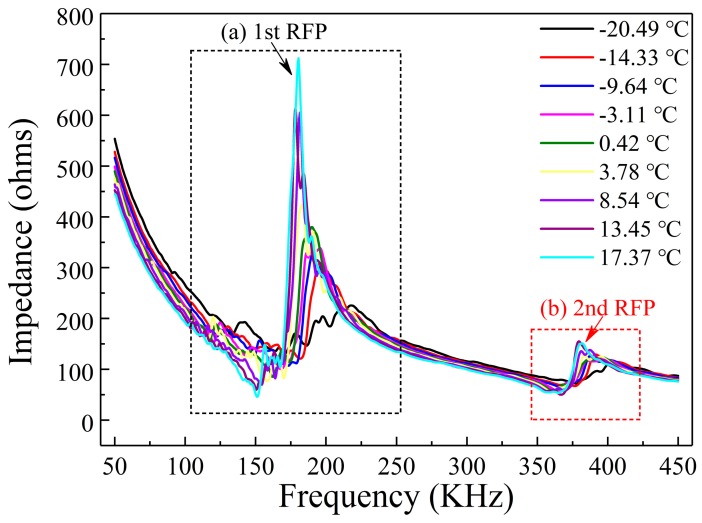
Impedance variation during the soil thawing process: (**a**) the first observed resonant frequency peak; (**b**) the second observed resonant frequency peak.

The variation of impedance signatures measured by the PZT-based transducer at different temperatures in the freezing process is shown in Figure 5. As can be seen from the figure, two resonance frequency peaks (RFP) appear in the frequency range of 0–450 KHz. The first observed resonance peak is located at about 175 kHz, and the second observed peak is located at around 375 kHz. It was found that as the temperature decreased, the resonance peak value of the impedance signature declined and the resonance frequency shifted to the right. The variation of the impedance signal with the temperature during the thawing process is shown in Figure 6. It can be seen from the figure that with increasing temperature, the magnitude of the resonance peak presented an upward trend and the resonance frequency shifted to the left. These results demonstrate that the impedance signature is very sensitive to changes in temperature. The trends of the resonance frequency peaks show a clear pattern with variation of the temperature, which reflects that the shift of the resonance frequency peak can serve as an indicator to monitor the soil freeze–thaw process. Therefore, the correlation between the resonance peaks in the EMI spectra and the temperature was analyzed; this is described below.

**Figure 7:**
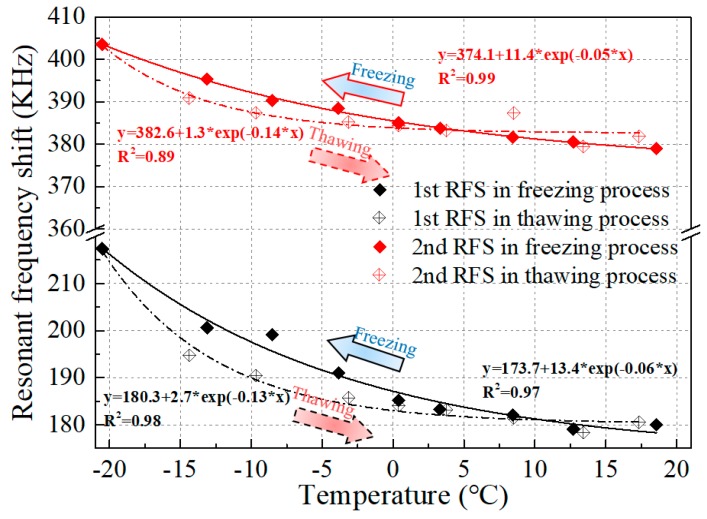
Resonant frequency shift (RFS) versus temperature.

**Figure 8:**
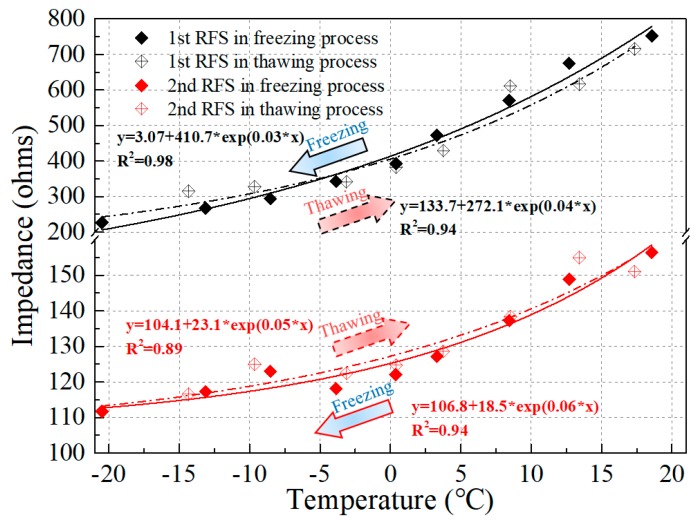
Variation of impedance at resonant frequencies with temperature.

The relationship between the resonant frequency and the temperature was obtained by comparing the changes in the resonant frequency of the impedance signal of the PZT transducer, and the fitted correlation curves between the two variables along with the corresponding values of *R^2^* are exhibited in Figure 7. It can be concluded that the resonant frequency shift has a negative correlation with the variation of the temperature. Both the first and second observed resonant frequencies declined as the temperature increased and went up as the temperature decreased. This is mainly attributed to the changes in the soil properties induced by the soil freezing and thawing. Besides this, the temperature itself also had some influence on the impedance signature captured by the piezoceramic-based sensor. As shown in Figure 8, a relationship between the magnitude of the resonance frequency peak and the temperature was also observed. It can be concluded that the magnitude of the resonance peak presents a positive correlation with the temperature. The peak value increased as the temperature increased in the thawing process and declined as the temperature dropped in the freezing process. The analysis above illustrates that the resonance peak shift is a very effective part of monitoring the soil freeze–thaw process. Even the hysteresis between the freezing and thawing paths can be monitored.

### 4.3. Active Sensing during the Freeze–Thaw Process

**Figure 9:**
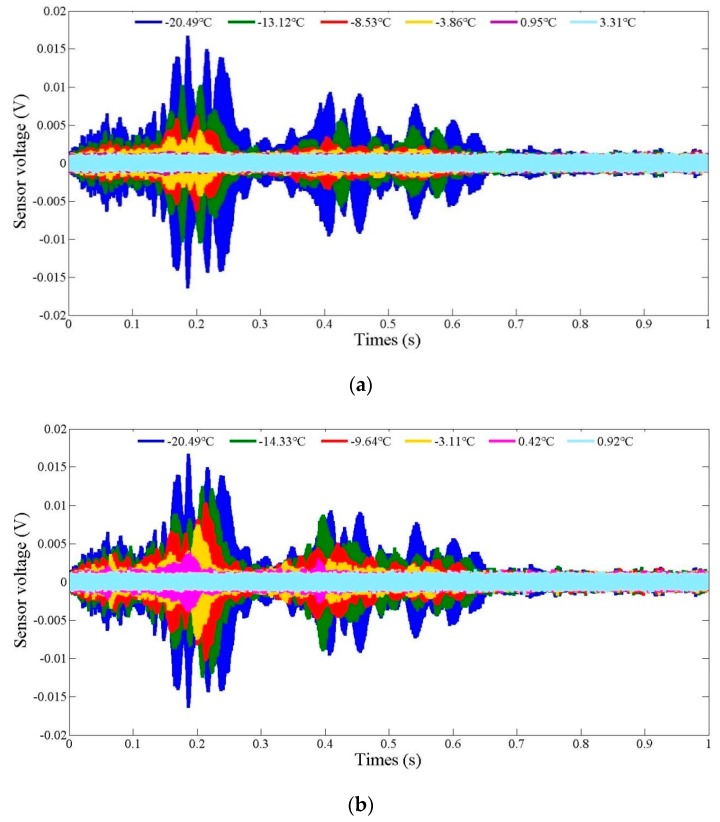
Variation of SA3 signals: (**a**) Freezing process; (**b**) Thawing process.

The time domain signal response of the PZT-based sensor during the soil freeze–thaw process is reflected in Figure 9. Each curve represents a one-second period of the sweep sine wave signal at a certain temperature. It can be seen from the figure that the amplitude of the signal presents an upward trend with decreasing temperature in the freezing process. This is mainly due to the fact that the stiffness of the soil specimen increased significantly as the soil began to harden in the freezing process, which resulted in a reduction in the signal energy dissipation. It is noteworthy that there was no signal at all before the temperature reached 0 °C and the soil began to freeze. These signals were exhibited in form of white noise, as shown in Figure 9. During the thawing process, the reverse results were observed, as shown in Figure 9b. The experiment results are in good agreement with those obtained in the studies by Kong et al. (2014) and Wang et al (2015) [90,91,92]. Thus, based on the above analysis, we can draw the conclusion that the active sensing signal is sensitive to the soil temperature change during the freeze–thaw process.

**Figure 10:**
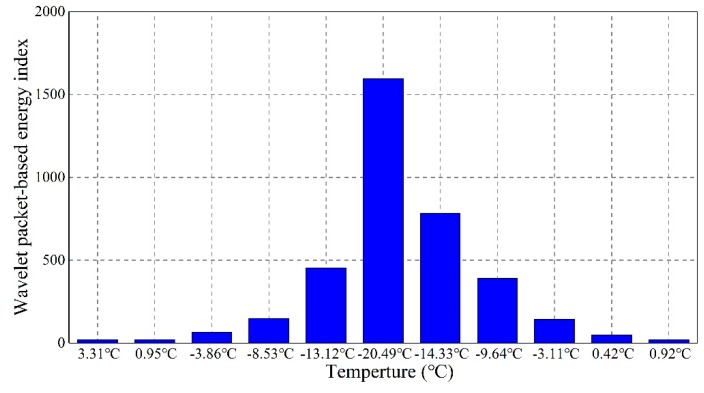
Variation of the wavelet packet energy index during the freeze–thaw process.

To fully explore the feasibility of the active sensing method to monitor the soil freeze–thaw process, the wavelet packet-based energy index of the active sensing signal was calculated using the wavelet packet analysis method. This can serve as a reliable approach to quantitatively evaluate the soil freeze–thaw status. The relationship between the wavelet packet-based energy index and the temperature is shown in Figure 10. As is shown, the calculated index went up sharply with an increased growth rate during the freezing process. As the soil was at the unfrozen state, that is, the temperature of the soil specimen was above 0 °C, the wavelet packet-based energy index was at a very low value, being close to 0. However, as the temperature dropped to the minimum value of about −20 °C, the index reached the highest value of more than 1500. During the defrosting process, the calculated index experienced a declining trend with increasing temperature and the declining rate gradually became lower and lower. This trend is quite similar to that of the resonance peak shift in the EMI spectra, as shown in Figure 7 and Figure 8, which validates that the EMI method is reliable for monitoring the soil freeze–thaw process and that the resonance peak shift can be adopted as an index to monitor the soil freeze–thaw process. Compared with the active sensing method, the EMI method has more advantages. Only one transducer is needed in the EMI method, and the results are directly reflected by the impedance analyzer without any complicated mathematical calculations. In addition, many low-cost systems have been developed to apply this approach [95]. Thus, it is expected that we will see more studies using the EMI method for monitoring the soil freeze–thaw process in related engineering projects.

## 5. Conclusions and Future Works

In this research, based on the electromechanical impedance (EMI) method and the active sensing method, three PZT-based smart aggregates were used to monitor the soil freezing–thawing process. In the EMI tests, it was noted that the impedance signal was very sensitive to the temperature change in the soil freeze–thaw process. In the freezing process, the resonance peak of the impedance signature shifted towards the right and in a downward direction. Meanwhile, in the thawing process, the magnitude of the resonance peak gradually went up and the resonance frequency declined. This illustrates that the resonance peak shift is can be effectively used to monitor the soil freeze–thaw process. Moreover, through the active sensing method, the recorded stress wave signal was also found to be sensitive to the temperature change. A significant increasing trend of the signal amplitude was acquired due to the enhancement of the soil stiffness in the freezing process, and a converse trend was observed in the thawing process. To conduct further research on the active sensing method used for monitoring the soil freeze–thaw process, analysis of the wavelet packet-based energy index of the stress wave signal was performed, and its correlation with the soil temperature was established by the wavelet packet analysis. The results reveal that the wavelet packet-based energy index can act as a reliable indicator to quantitatively estimate the soil freeze–thaw process. Thus, this experimental research demonstrated that it is feasible to monitor the soil freezing–thawing status by using a PZT-based electromechanical method. In future research on this topic, factors that may affect the experimental results, such as the soil moisture, type, and repeated freezing–thawing cycles, will be investigated. Moreover, in future works, some statistical metrics such as the root-mean-square deviation (RMSD), the mean absolute percentage deviation (MAPD), and the cross-correlation coefficient (CC) will also be employed as indicators for the quantitative evaluation of the changes in the impedance signals.
